# The Rabies Concert: Rising towards the Last Waltz?

**DOI:** 10.3390/tropicalmed6030124

**Published:** 2021-07-08

**Authors:** Charles E. Rupprecht

**Affiliations:** LYSSA LLC, Cumming, GA 30040, USA; charleserupprechtii@gmail.com

**Keywords:** elimination, encephalitis, lyssavirus, neglected tropical disease, One Health, prophylaxis, rabies, vaccination, virus, zoonosis

Apologies for the brief intermission imposed by the pandemic, between the opening piece of our Special Issue “Lyssaviruses and Rabies: Prevention, Control and Elimination” and this closing act of commentary. Figuratively speaking, there is an old band back in town, tuning their delicate instruments for a final performance—the global elimination of human rabies from dogs (GEHRD), with the anticipation of culmination to zero cases by the year 2030 (ZBT). This virological melody should sound somewhat familiar, because the biomedical players of multiple generations have been on this prevention and control stage for hundreds of years. Encores are anticipated, and some variation on this particular tune may be played for centuries to come, just not as loudly and perhaps less well-attended, because rabies could be managed, but it is not a candidate for eradication (despite some critical reviews). Simply stated, reservoirs abound among wildlife. Attached for your scientific amusement is a rather short, focused playbill to stir the passions and delight the imagination with flights of epidemiological fancy, transforming a seemingly difficult zoonotic arrangement to a masterful One Health concert. 

More than a dozen cohesive papers have been published upon peer review acceptance in this Special Issue (i.e., nine original papers, one case report, and three review papers are included). Collectively, they include more than 40 citations and 17,000 public views to date. Each manuscript contributes to a much better understanding of this zoonosis and to progress concerning improvements for rabies surveillance, prevention, control, and selective elimination. Rather than one limited tune, these papers cover a diversity of topics important to the field.

To begin, the ability to diagnose a rabies case began in the early twentieth century, and has progressed greatly over the past century. Yet, diagnostic capabilities are still lacking in many parts of the developed world, particularly in Africa and Asia. Without a diagnosis, there is no case confirmation, no ability to properly perform a risk assessment on exposed humans and animals, and no adherence to laboratory-based surveillance or true evaluation of vaccination programs. Point-of-care lateral flow tests have improved over the last several decades to meet this challenge. Regrettably, sensitivity and specificity vary greatly by different products, as shown by Klein et al. [1].

Lackluster is not a term applied to our specialized vaccine instruments, human or veterinary. Vero cell vaccine is one modern biologic that has proved its mettle regarding purity, potency, safety, and efficacy for both pre- (PrEP) and post-exposure prophylaxis (PEP), as reviewed by Moulenat et al. [2]. Moreover, such vaccines may be applied in dose-sparing schedules via the intradermal route with health economic benefits, as shown by Kundu et al. [3]. Besides human vaccines, rabies immune globulin (RIG), infiltrated into and around wounds, is a key part of PEP, providing a bridge of passive immunity immediately before the induction of virus-neutralizing antibodies actively from vaccination. Exposed patients have succumbed in the absence of RIG. If RIG is so critical during PEP, why is it omitted? In part, RIG is expensive and largely unavailable for clinics that attend to hundreds of patients per day. As an alternate, monoclonal antibodies (MAbs) were developed during the 1970s, and have finally come to fruition with the first licensed products in India. Additional MAb cocktails with an adequate breadth of neutralizing activity are necessary for the marketplace (both human and animal), as described by Chao et al. [4].

Besides human PEP, the vanguard for superiority in the GEHRD is mass canine vaccination programs. Excellent examples are provided by Athingo et al. [5] for rural areas in southern Africa, as well as on a sub-continental level by Gibson et al. [6]. Although products for animals meet stringent criteria for regulatory approval as human biologics, no vaccine meets 100% efficacy or safety. For example, Arega et al. [7] reported on a rather curious phenomenon of temporal mortality observed in vaccinated puppies, that defies simple explanation to date. Besides the need for continued surveillance and enactment of a vaccine adverse event reporting monitor, product integrity remains a criticality often ignored. As most canine vaccination campaigns occur in rather hot, tropical environments, thermostability is a concern. Rising to creative heights, Lungelo et al. [8] developed an opportunity for developing cooling devices for vaccines, created at a local level in Tanzania.

In addition to human and canine applications, the vaccination of free-ranging wildlife begun during the 1970s. Tizzani et al. [9] described their success in rabies vaccination of foxes in eastern Europe, while Robardet et al. [10] review the remarkable benefits of the oral vaccination program at a grand scale throughout Europe. Individual and parenteral vaccination of owned, captive, or trapped animals, domestic or wild, is less challenging than those that are unavailable or take flight at the approach of humans. In a second communication, Gibson et al. [11] reported on the oral vaccination concept applied to community dogs in Goa, India, which recently gained a major accolade as a previously enzootic area to gain freedom from rabies. Such innovations should benefit the GEHRD in a few years.

It might be exciting to culminate with a standing ovation and the veritable hair of the dog stanza now, but not quite yet; rounding out this ensemble is a nod to the reality of perpetuation by a quintessential disease of nature. More than 17 lyssaviruses have been described to date. All cause rabies. Most reside among bats, with session players comprised of several wild carnivores, non-human primates, and other mammalian groups still awaiting recognition. Although different lyssavirus species are culpable in the Old World, true bat rabies, caused by the ‘type species’ for the Genus, rabies virus, is limited to the New World. Many variants have been described. This creates a bit of a problem for those localities that have already achieved the GEHRD locally. Morgan et al. [12] exemplified the dilemma by modeling the existence of bat rabies in the Caribbean, a region long felt to be “rabies-free”, largely because of a dearth of laboratory-based surveillance, yet dues are payable to master a certain song with acclaim. Finally, no related song book would be complete without a bow to vampires, the divas of the bat rabies world. No other bat species carries the dual cachet and pedigree—the only mammal subsisting entirely on blood and the only mammal that is a routine viral transmitter via the act of feeding. Rabid vampire bats may have prospered as long ago as the Pleistocene, with swelling ranks since the time of the conquistador and their free meal on the hoof via introduced livestock. Despite their biological novelty, broad distribution from Mexico to Argentina, and historical burden, many important biological facets as a reservoir remain unknown. Combined captive and field studies may shed light upon some of these aspects, perhaps leading to better management techniques. As related by Cárdenas-Canales et al. [13], curious insights into the pathobiology of rabies virus, and the ability of vampire bats to respond in kind, were revealed by the unintended consequences from a laboratory outbreak—there and back again.

Time for a final curtain call? Unfortunately, true progress on the prevention and control of all NTDs, such as rabies, remains a challenge, even before a pandemic. As shown by this special issue, salient tools to meet the ZBT goal are available, at least since the last century. Current research shows that additional inroads are still possible towards a basic understanding of one of the world’s oldest zoonoses, as communicated by dedicated workers in the field. Future work will continue to aid the GEHRD task that is well underway, which will not be simple, rapid, or inexpensive. Clearly, while hope springs eternal that one’s dancing shoes will fit better another night, the time to rise to the beat is now. Waiting to begin until the arrival of another magical, outside mystery tour is simply not a reliable option, because, ultimately, “... no one else is coming ...” to this timely event; you are the composer, band leader, performer, and dancer alike, as the show must go on and the song remains the same.

## Data Availability

Not applicable.

## References

[1] Klein A., Fahrion A., Finke S., Eyngor M., Novak S., Yakobson B., Ngoepe E., Phahladira B., Sabeta C., De Benedictis P. (2020). Further Evidence of Inadequate Quality in Lateral Flow Devices Commercially Offered for the Diagnosis of Rabies. Trop. Med. Infect. Dis..

[2] Moulenat T., Petit C., Bosch Castells V., Houillon G. (2020). Purified Vero Cell Rabies Vaccine (PVRV, Verorab^®^): A Systematic Review of Intradermal Use Between 1985 and 2019. Trop. Med. Infect. Dis..

[3] Kundu B.K., Meshram G.G., Bhargava S., Meena O. (2019). Cost Savings of Using Updated Thai Red Cross Intradermal Regimen in a High-Throughput Anti-Rabies Clinic in New Delhi, India. Trop. Med. Infect. Dis..

[4] Chao T.-Y., Zhang S.-F., Chen L., Tsao E., Rupprecht C.E. (2020). In Vivo Efficacy of SYN023, an Anti-Rabies Monoclonal Antibody Cocktail, in Post-Exposure Prophylaxis Animal Models. Trop. Med. Infect. Dis..

[5] Athingo R., Tenzin T., Shilongo A., Hikufe E., Shoombe K.K., Khaiseb S., van der Westhuizen J., Letshwenyo M., Torres G., Mettenleiter T.C. (2020). Fighting Dog-Mediated Rabies in Namibia—Implementation of a Rabies Elimination Program in the Northern Communal Areas. Trop. Med. Infect. Dis..

[6] Gibson A.D., Wallace R.M., Rahman A., Bharti O.K., Isloor S., Lohr F., Gamble L., Mellanby R.J., King A., Day M.J. (2020). Reviewing Solutions of Scale for Canine Rabies Elimination in India. Trop. Med. Infect. Dis..

[7] Arega S., Conan A., Sabeta C.T., Crafford J.E., Wentzel J., Reininghaus B., Biggs L., Leisewitz A.L., Quan M., Toka F. (2020). Rabies Vaccination of 6-Week-Old Puppies Born to Immunized Mothers: A Randomized Controlled Trial in a High-Mortality Population of Owned, Free-Roaming Dogs. Trop. Med. Infect. Dis..

[8] Lungelo A., Hampson K., Bigambo M., Kazwala R., Lankester F. (2020). Controlling Human Rabies: The Development of an Effective, Inexpensive and Locally Made Passive Cooling Device for Storing Thermotolerant Animal Rabies Vaccines. Trop. Med. Infect. Dis..

[9] Tizzani P., Fanelli A., Potzsch C., Henning J., Šašić S., Viviani P., Hrapović M. (2020). Wildlife and Bait Density Monitoring to Describe the Effectiveness of a Rabies Vaccination Program in Foxes. Trop. Med. Infect. Dis..

[10] Robardet E., Bosnjak D., Englund L., Demetriou P., Rosado Martín P., Cliquet F. (2019). Zero Endemic Cases of Wildlife Rabies (Classical Rabies Virus, RABV) in the European Union by 2020: An Achievable Goal. Trop. Med. Infect. Dis..

[11] Gibson A.D., Mazeri S., Yale G., Desai S., Naik V., Corfmat J., Ortmann S., King A., Müller T., Handel I. (2019). Development of a Non-Meat-Based, Mass Producible and Effective Bait for Oral Vaccination of Dogs against Rabies in Goa State, India. Trop. Med. Infect. Dis..

[12] Morgan C.N., Wallace R.M., Vokaty A., Seetahal J.F.R., Nakazawa Y.J. (2020). Risk Modeling of Bat Rabies in the Caribbean Islands. Trop. Med. Infect. Dis..

[13] Cárdenas-Canales E.M., Gigante C.M., Greenberg L., Velasco-Villa A., Ellison J.A., Satheshkumar P.S., Medina-Magües L.G., Griesser R., Falendysz E., Amezcua I. (2020). Clinical Presentation and Serologic Response during a Rabies Epizootic in Captive Common Vampire Bats *(Desmodus rotundus)*. Trop. Med. Infect. Dis..

